# Epithelioid pleural mesothelioma successfully treated with perioperative immunotherapy: a case report

**DOI:** 10.1186/s44215-024-00157-3

**Published:** 2024-06-11

**Authors:** Gaku Yamazaki, Aki Fujiwara-Kuroda, Jun Muto, Hideki Ujiie, Masato Aragaki, Megumi Furuta, Sakurako Ohno, Kanako C. Hatanaka, Yutaka Hatanaka, Yoshihiro Matsuno, Tatsuya Kato

**Affiliations:** 1 Department of Thoracic Surgery, Hokkaido University Hospital, Kita-Ku, Sapporo, Hokkaido, N14W5, 060-8648 Japan; 2 Department of Respiratory Medicine, Faculty of Medicine, Hokkaido University, Sapporo, Japan; 3 Department of Surgical Pathology, Hokkaido University Hospital, Sapporo, Japan; 4 Center for Development of Advanced Diagnostics, Hokkaido University Hospital, Sapporo, Japan

**Keywords:** Pleural mesothelioma, Immune checkpoint inhibitor, Pleurectomy/decortication, Salvage surgery

## Abstract

**Background:**

Pleural mesothelioma, characterized by a dismal prognosis even with multimodal therapy, has seen emerging interest in immune checkpoint inhibitors (ICIs) due to their demonstrated efficacy. Here, we present a case of epithelioid-type pleural mesothelioma with chest wall invasion treated with definitive ICI therapy, resulting in a remarkable pretreatment effect.

**Case presentation:**

A 46-year-old man was diagnosed with an abnormal chest shadow on a medical check, and a computed tomography scan showed pleural thickening at the dorsal right upper chest wall. One of the nodules was suspected to have invaded the chest wall. A needle biopsy revealed epithelioid-type pleural mesothelioma. After five cycles of nivolumab plus ipilimumab, he underwent right pleurectomy/decortication (P/D). Pathological examination revealed a significant treatment effect, showing numerous lymphocytes infiltrating the tumor nodule and viable tumor cells localized at approximately 6 mm.

**Conclusion:**

Although further accumulation of cases is required to evaluate the effectiveness and case selection, P/D after immunotherapy is a useful curative treatment option for advanced pleural mesothelioma.

## Background

Although pleural mesothelioma has a poor prognosis even with multidisciplinary treatment, The efficacy of immune checkpoint inhibitors (ICIs) has recently been demonstrated.

Herein, We report a case in which definitive ICI treatment was performed for epithelioid-type pleural mesothelioma with chest wall invasion, and a remarkable pretreatment effect was observed

## Case presentation

**Fig. 1:**
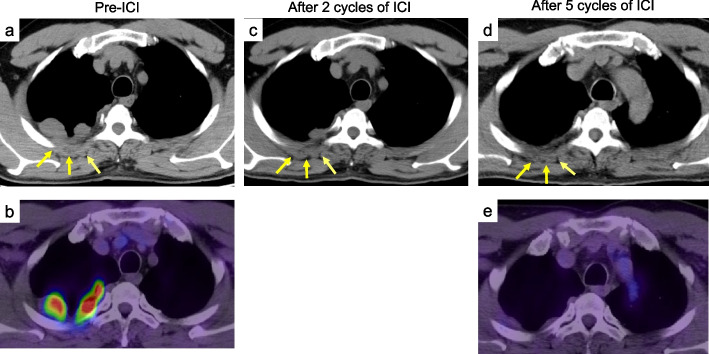
Dramatic preoperative effects of immune checkpoint inhibitors (ICI). Chest CT and ^18F−^FDG-PET findings showed pleural thickening of the dorsal right upper lobe and multiple nodules, including a nodule with a maximum diameter of 34 mm, which was suspected to have invaded the chest wall (cT4) (arrows) (**a**), which showed an abnormal SUV uptake value of 19.9 (**b**). After two cycles of ICI, the tumor has shrunk, and the part invading the chest wall has become less distinct (**c**). After five cycles of ICI, further tumor regression (**d**) and abnormal SUV uptake (**e**) were not observed

A 46-year-old man was diagnosed with an abnormal shadow on chest radiography during a medical checkup. The patient has a smoking history of 20 pack-years and was environmentally exposed to asbestos for 4 years between the ages of 22 and 25 while working at a railway yard (asbestos present in walls/ceilings of the workplace). He presented with right back pain, and computed tomography revealed pleural thickening at the dorsal right upper lesion and multiple nodules. One of the nodules was suspected of invading the chest wall, with a maximum diameter of 34 mm (Fig. 1a). A needle biopsy revealed an epithelioid mesothelioma. ^18F−^fluorodeoxyglucose positron emission tomography (FDG-PET) showed an abnormal standardized uptake value of 19.9 (Fig. 1b). Nivolumab (Nivo) 360 mg/body every 3 weeks plus ipilimumab (Ipi) 1 mg/kg every 6 weeks as a definitive treatment was planned because of clinical T4N0M0 (stage IIIB) according to the World Health Organization classification (5th Edition). After two cycles of ICI, the tumor has shrunk, and the part invading the chest wall has become less distinct (Fig. 1c). After five cycles, the tumor dramatically shrunk (Fig. 1d), and the FDG-PET scan showed no abnormal uptake; therefore, it was judged to be resectable (Fig. 1e). The patient underwent a right pleurectomy/decortication (P/D) approximately 4 months after the initial diagnosis.

**Fig. 2:**
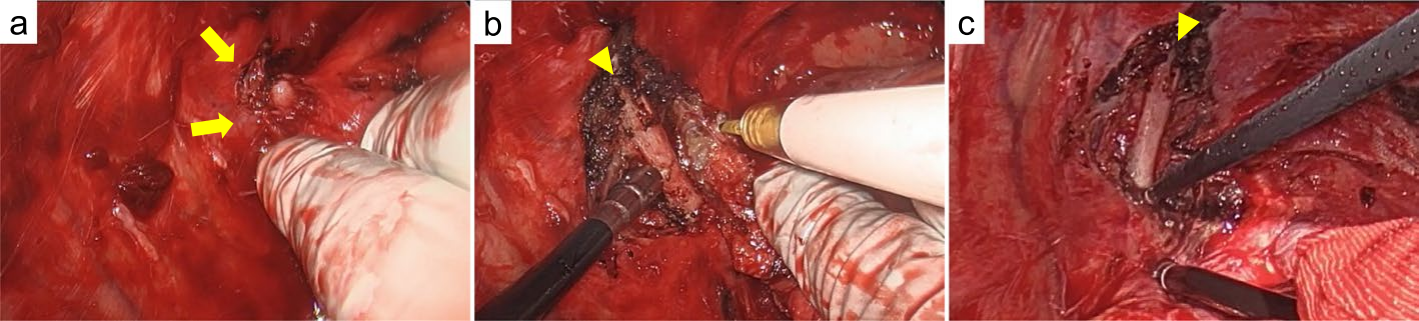
Intraoperative findings. Strong fibrosis was observed between the 2nd and 3rd intercostal spaces (arrows), likely where the tumor was present before ICI treatment (**a**). Although direct invasion was not observed in the 2nd rib (arrowhead), it was noted to be embedded in the intercostal muscles, and these areas were resected combined with part of the intercostal muscles (**b**, **c**)

Surgery was performed via right posterolateral thoracotomy through the 7th intercostal space. During surgery, strong fibrosis was observed between the 2nd and 3rd intercostal spaces, likely where the tumor was present before ICI treatment (Fig. 2a). Although direct invasion was not observed in the 2nd rib, it was noted to be embedded in the intercostal muscles, and these areas were resected combined with part of the intercostal muscles (Fig. 2b and c). No obvious invasion was observed in other areas, and except for some thickening of the visceral pleura of the lung in the upper lobe, the other part of the pleura appeared normal and was dissected in the usual manner. No evidence of pericardial or diaphragmatic invasion was also observed.

**Fig. 3:**
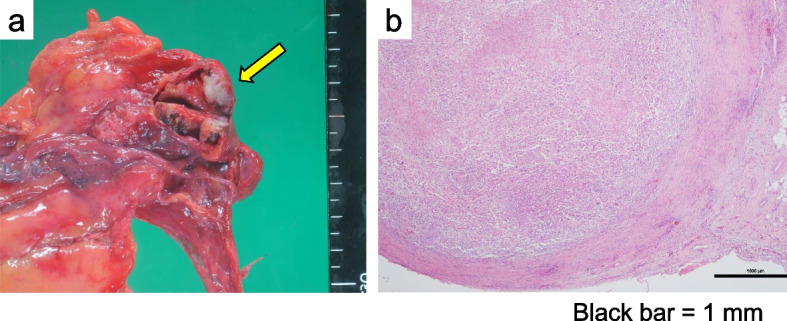
Macroscopic findings. The resected specimens show widespread nodular fibrous thickening of the pleura, presumed to be a posttreatment scar (arrow) (**a**). Pathological evaluation revealed that viable tumor cells were localized at approximately 6 mm in the right upper dorsal nodule (ypT1) (**b**)

**Fig. 4:**
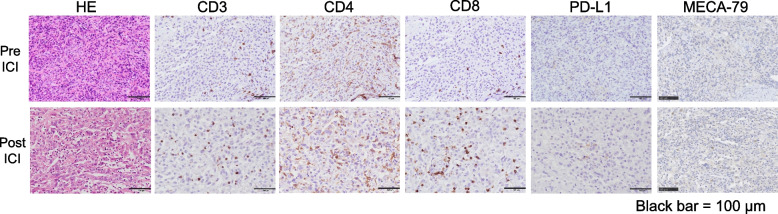
Pathological examination and immunohistochemical study. The intercellular space between tumor cells increased, and CD3, CD4, and CD8 staining showed the infiltration of numerous lymphocytes into the tumor nodule after immunotherapy. Both PD-L1 and MECA-79 staining were negative in both the biopsy and resected specimens

Pathological findings showed multifocal, irregularly shaped, fibrous thickening throughout the resected pleura. Because viable tumor cells were not identified in these lesions, they were presumed to be post-treatment scars. Residual tumor cells were not found at the resected margins, including the intercostal muscles. A solitary viable tumor nodule approximately 6 mm in diameter was found only in the right upper dorsal pleura (ypT1, Fig. 3). Furthermore, the tumor cells were more incohesively arranged than those seen in the pretreatment biopsy, accompanied by an obviously greater degree of CD3-, CD4-, or CD8-positive mononuclear cell infiltration inside the tumor nests after immunotherapy (Fig. 4). Programmed death-ligand 1 (PD-L1) and MECA-79 (epitope of sialyl 6-sulfo Lewis X determinant) expressions in tumor cells were immunohistochemically negative in both the pretreatment biopsy and resected specimens (Fig. 4).

The postoperative course was uneventful, and the patient was discharged on postoperative day 19. He was restarted on ICI 9 weeks after surgery and continued to receive it every 3 weeks. He was followed up without recurrence 14 months after surgery.

## Discussion and conclusions

Pleural mesothelioma has a poor prognosis even with multimodal treatment; however, the combination ICI therapy with Nivo plus Ipi was reported to prolong overall survival in the CheckMate-743 trial [1]. In terms of the pathological complete response (pCR), the overall pCR rate with ICI therapy in CheckMate816 was generally around 24% in non-small cell lung cancer (NSCLC) [2]. A subsequent meta-analysis also reported a rate of 28.1%, suggesting that approximately one-fourth of patients achieve pCR in NSCLC [3]. On the other hand, in pleural mesothelioma cases where induction therapy using ICI followed by surgical resection is not covered by insurance and there have been no reports of clinical trials using preoperative induction therapy with ICIs so far unlike NSCLC, making it difficult to accurately compare pCR rates. Complete resolution of FDG uptake within the tumor volume indistinguishable from surrounding normal tissue was determined as a complete metabolic response (CMR). It was considered that both FDG-PET and CT criteria are accurate for response evaluation of ICI therapy and prediction of pleural mesothelioma prognosis. The major three FDG-PET criteria (EORTC, PERCIST, imPERCIST) judged a greater percentage of patients (16.7%) as CMR [4]. Although it is difficult to make a simple comparison because it involves pathological evaluation and visual comparison, NSCLC seems to have a slightly higher response rate.

Although it has shown efficacy as a primary treatment for unresectable pleural mesothelioma, few studies have reported its use in combination with surgical treatments. In our facility, a total of 28 cases have received ICI therapy, including 11 cases of Ipi plus Nivo and 19 cases of Nivo monotherapy. Among these, there were three cases where surgery was performed after ICI administration, with only one case undergoing a pleurectomy/decortication (present case), and in two cases of Nivo monotherapy, local excision of the recurrent sites was performed. In this report, a patient with epithelioid pleural mesothelioma in which the tumor was suspected of invading the chest wall was successfully treated with ICI treatment, and P/D was performed as a complete resection owing to a remarkable pretreatment effect. The patient tolerated the ICI treatment without any side effects; therefore, surgery was performed without any perioperative complications. Multidisciplinary treatments combining definitive ICI followed by salvage surgery have shown promise as curative treatments for advanced or unresectable pleural mesotheliomas. The patient also continued ICI treatment after surgery, although the use of ICI postoperatively remains controversial.

ICI was reported to be effective regardless of the histological type, and a study reported a patient with unresectable sarcomatous-type pleural mesothelioma treated with ICI followed by salvage P/D with partial response (PR) [5]. Another report revealed that salvage P/D for interstitial pneumonia after ICI treatment for unresectable epithelioid-type pleural mesothelioma showed no perioperative ICI-associated complications [6].

However, adverse events of ICI therapy, including interstitial lung disease and endocrine disorders, should be considered immune-related adverse events (irAE) [7]. Once this occurs, both immunosuppressive agents and high-dose steroid coverage are required during the perioperative period, which is presumed to be important because of the prolonged wound healing and increased risk of infection. As anticipated, more patients will receive definitive ICI therapy in the future, and detailed protocols will be required for steroid coverage, including the number and duration of irAEs.

In the present case, pathological findings confirmed that definitive immunotherapy had a significant treatment effect. Immunostaining for CD3, CD4, and CD8 revealed numerous lymphocytes infiltrating the tumor nodules. A systematic review of ICI therapy for pleural mesothelioma revealed that PD-L1 expression in tumor cells was not a sufficient predictor of treatment response, unlike other cancer types [8]. Although PD-L1 expression in tumor cells was not high in this case, ICI showed a marked therapeutic effect. The MECA-79-reactive antigen is closely associated with carbohydrate ligands for L-selection (e.g., CD34, GlyCAM-1, and MAdCAM-1), which are highly expressed on high endothelial venules (HEV) during lymphoid tissue inflammation [9]. MECA-79 + tumor-associated HEVs (TA-HEVs) are frequently found in human tumors in CD3 + T cell-rich areas or CD20 + B cell-rich tertiary lymphoid structures. TA-HEVs have been proposed to play an important role in lymphocyte entry into tumors, a process essential for successful antitumor immunity and lymphocyte-mediated cancer immunotherapy using ICI [6]. However, in the present case, no obvious MECA-79 staining was observed pre- or post-ICI treatment. A novel marker predicting the effect of ICIs on pleural mesothelioma is urgently needed, and further clinical studies are necessary.

In conclusion, this case provides pathological evidence of the efficacy of immunotherapy and indicates that P/D after immunotherapy is a useful curative treatment option for advanced pleural mesothelioma. The number of cases in which ICI is performed as a definitive treatment initially followed by salvage surgery is expected to increase in the future, and further accumulation of cases is needed to evaluate its effectiveness and case selection.

## Data Availability

Data sharing is not applicable to this article, as no datasets were generated or analyzed during the current study.

## Abbreviations

FDG-PET: ^18F^^−^fluorodeoxyglucose positron emission tomography
irAE: Immune-related adverse event
HEV: High endothelial venules
ICI: Immune checkpoint inhibitors
P/D: Pleurectomy/decortication
PD-L1: Programmed death-ligand 1
TA-HEVs: Tumor-associated HEVs
